# Supplementary material for: Quantification of Zonisamide in Dried Blood Spots and Dried Plasma Spots by UPLC–MS/MS: Application to Clinical Practice
Source: Molecules. 2022 Jul 31;27(15):4899. doi: 10.3390/molecules27154899 (PMC9369825; doi:10.3390/molecules27154899)
Supplement: Supplementary file 1 [file molecules-27-04899-s001.zip › molecules-1813030-supplementary.pdf]

Table S1. Stability testing.

		DPS		DBS	
		**low QC**	**high QC**	**low QC**	**high QC**
**nominal concentration, μg/ mL**		**0.750**	**38.0**	**0.375**	**19.0**
**short term stability**	**<bias>, %**	-1.42	-4.75	7.06	3.77
	**RSD, %**	2.52	3.53	1.12	7.10
**long term stability**	**<bias>, %**	7.78	4.31	8.65	7.01
	**RSD, %**	3.12	3.94	3.58	4.11
**auto sampler stability**	**<bias>, %**	7.59	7.23	2.13	3.11
	**RSD, %**	4.89	5.73	2.36	5.05
**n**		3	3	3	3

<bias> - percentage bias presents deviation of mean ZNS concentration determined at certain QC level after applied storage conditions from the nominal concentration; ((mean ZNS concentration – nominal concentration)/nominal concentration) × 100; calculated value should be ± 15%.; RSD – percentage coefficient of variation for calculated bias at certain QC level; n – number of replication at certain QC level.

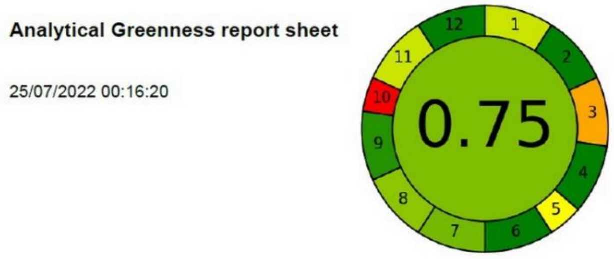

Criteria	Score	Weight
1. Direct analytical techniques should be applied to avoid sample treatment.	0.6	2
2. Minimal sample size and minimal number of samples are goals.	1.0	2
3. If possible, measurements should be performed in situ.	0.33	2
4. Integration of analytical processes and operations saves energy and reduces the use of reagents.	1.0	2
5. Automated and miniaturized methods should be selected.	0.5	1
6. Derivatization should be avoided.	1.0	2
7. Generation of a large volume of analytical waste should be avoided, and proper management of analytical waste should be provided.	0.77	2
8. Multi-analyte or multi-parameter methods are preferred versus methods using one analyte at a time.	0.72	2
9. The use of energy should be minimized.	0.93	2
10. Reagents obtained from renewable sources should be preferred.	0.0	1
11. Toxic reagents should be eliminated or replaced.	0.6	2
12. Operator's safety should be increased.	1.0	2

Figure S1. Greenness of the proposed UHPLC-MS/MS method - Analytical greenness report with overall green Scheme 12. principles of the Green Analytical Chemistry.
